# Real-time two- and three-dimensional imaging of monocyte motility and navigation on planar surfaces and in collagen matrices: roles of Rho

**DOI:** 10.1038/srep25016

**Published:** 2016-04-28

**Authors:** Robert Bzymek, Markus Horsthemke, Katrin Isfort, Simon Mohr, Kerstin Tjaden, Carsten Müller-Tidow, Marlies Thomann, Tanja Schwerdtle, Martin Bähler, Albrecht Schwab, Peter J. Hanley

**Affiliations:** 1 Institut für Physiologie II, Westfälische Wilhelms-Universität, 48149 Münster, Germany; 2 Institut für Molekulare Zellbiologie, Westfälische Wilhelms-Universität, 48149 Münster, Germany; 3 Molekulare und Vaskuläre Kardiologie, Universitätsklinikum Münster, 48129 Münster, Germany; 4 Hämatologie und Onkologie, Universitätsklinikum Halle, 06120 Halle, Germany; 5 Analytical and Environmental Sciences Division, King’s College London, United Kingdom; 6 Institut für Ernährungswissenschaft, Universität Potsdam, 14558 Nuthetal, Germany

## Abstract

We recently found that macrophages from RhoA/RhoB double knockout mice had increased motility of the cell body, but severely impaired retraction of the tail and membrane extensions, whereas RhoA- or RhoB-deficient cells exhibited mild phenotypes. Here we extended this work and investigated the roles of Rho signaling in primary human blood monocytes migrating in chemotactic gradients and in various settings. Monocyte velocity, but not chemotactic navigation, was modestly dependent on Rho-ROCK-myosin II signaling on a 2D substrate or in a loose collagen type I matrix. Viewed by time-lapse epi-fluorescence microscopy, monocytes appeared to flutter rather than crawl, such that the 3D surface topology of individual cells was difficult to predict. Spinning disk confocal microscopy and 3D reconstruction revealed that cells move on planar surfaces and in a loose collagen matrix using prominent, curved planar protrusions, which are rapidly remodeled and reoriented, as well as resorbed. In a dense collagen type I matrix, there is insufficient space for this mode and cells adopt a highly Rho-dependent, lobular mode of motility. Thus, in addition to its role in tail retraction on 2D surfaces, Rho is critical for movement in confined spaces, but is largely redundant for motility and chemotaxis in loose matrices.

The two principal forces driving cell motility are actin polymerization and actomyosin contraction, mediated by (nonmuscle) class II myosins^1^. These forces are coordinated temporally and spatially by the on-off activity of membrane-anchored Rho GTPases: activated Rac and Cdc42 subfamilies induce actin polymerization and membrane protrusions, whereas activated members of the Rho subfamily of GTPases (RhoA, RhoB and RhoC) increase actomyosin activity via Rho kinases (ROCK1 and ROCK2)^2^. In addition to coordinating cell shape changes, migrating cells may use integrins (transmembrane adhesion molecules) to attach to the extracellular matrix or metallopeptidases (proteolytic enzymes) to degrade matrix components. Thus, various modes of migration can be generated by combinations of membrane protrusions, contractions, adhesions and proteolytic activity.

One of the defining features of any mode of cell migration is the nature of the protrusive structure at the front end. Most cell types use actin polymerization-driven lamellipodial (flat, sheet-like) protrusions to move on a 2D (two-dimensional) surface, whereas, in a 3D environment, cells may use either actin polymerization-driven structures (filopodia and pseudopodia) or Rho-dependent, actomyosin-driven structures (blebs and lobopodia).

Leukocytes are generally thought to move in a nonproteolytic (but see Van Goethem *et al*.^3^), amoeboid-like fashion, which is a loosely defined term, but implies that the cells generate actin polymerization-driven protrusions in the direction of movement, as exemplified by *Dictyostelium* amoebae^4^. Rho subfamily member A (RhoA) is a key regulator of cytoskeletal dynamics in cells and is implicated in at least two aspects of amoeboid-like migration, retraction of the trailing edge on 2D surfaces and squeezing of the nucleus (principal geometrically limiting factor^5^) through narrow spaces^6^.

The roles of the Rho subfamily in cell migration have been widely studied^3^,^6^,^7^,^8^,^9^,^10^,^11^,^12^, and it has become clear that the relative importance of Rho depends on cell type, mode of migration and the 2D or 3D microenvironment. In an elegant model, Petrie *et al*.^10^ proposed that fibroblasts read the extracellular matrix elasticity and can switch between low RhoA-ROCK-dependent, lamellipodial (nonlinear elastic matrix) and high RhoA-ROCK-dependent, lobopodial (linear elastic matrix) modes of migration (see also Friedl *et al*.^13^). Although RhoA is considered to be the predominant Rho subfamily member involved in cell motility, we found in a knockout mouse study^14^ that RhoB compensates for loss of RhoA, such that macrophages lacking either RhoA or RhoB had mild phenotypes, whereas cells lacking both RhoA and RhoB exhibited profound phenotypes, characterized by highly branched morphologies during random migration (largely due to impaired lamellipodial retractions) and exceedingly long trailing ends during directed migration. Chemotaxis efficiency was not impaired in macrophages lacking RhoA and RhoB (as well as, by default, RhoC), and, surprisingly, the velocity of the cell body, as well as *in vivo* monocyte/macrophage recruitment, were increased^14^. In the current study, we extended this work and used well-documented inhibitors to elucidate the roles of Rho-ROCK-myosin II signaling in the motility and chemotaxis of human monocytes on 2D surfaces and in both loose and dense (fibrillar) collagen type I matrices. We used two approaches, contrast and/or epi-fluorescence microscopy, which allowed time-lapse imaging for long durations (>10 h), and sequential spinning disk confocal microscopy, which allowed high-resolution 3D reconstruction of cells in motion, albeit in a shorter time span (<30 min).

## Results

### Expression of Rho subfamily GTPases and roles of ROCK-myosin II signaling in monocyte motility and chemotaxis on a 2D surface

**Figure 1:**
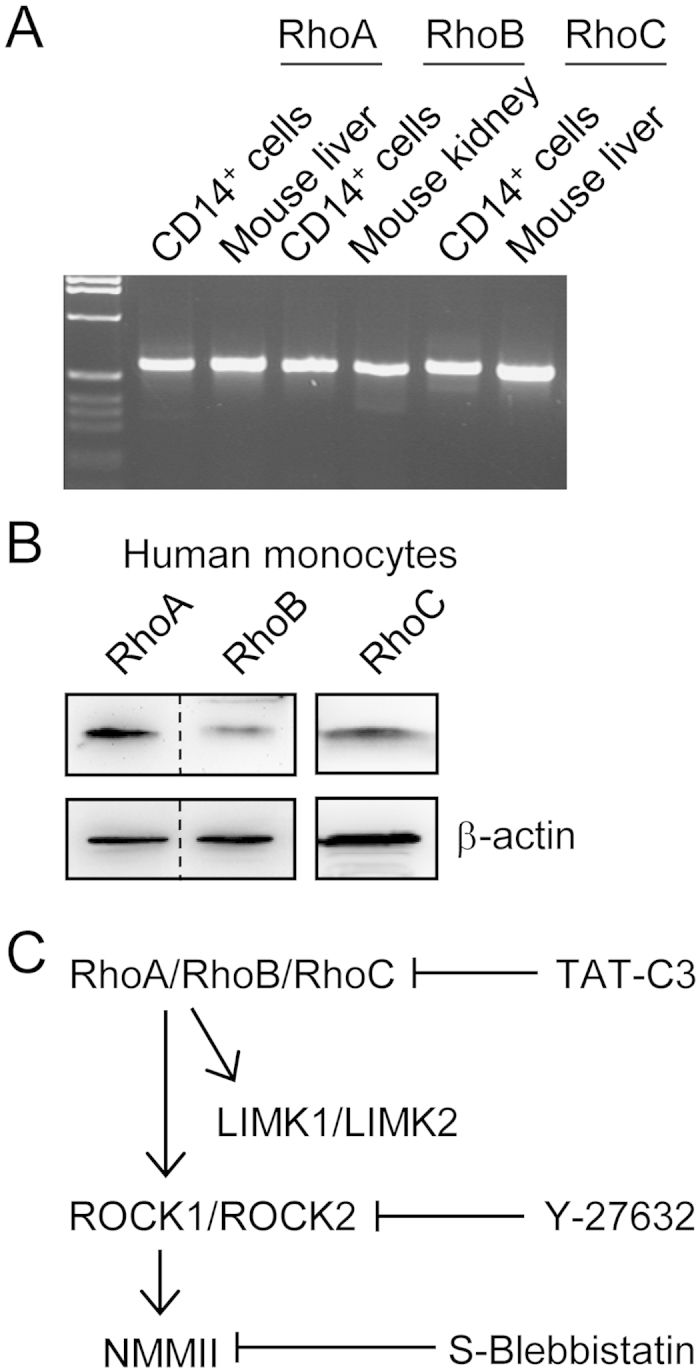
Rho subfamily expression and inhibitors of Rho-ROCK-myosin II signaling. (**A**) mRNA for RhoA, RhoB and RhoC could be detected by RT-PCR using RNA extracted from purified human CD14^+^ cells (monocytes). (**B**) Detection of RhoA, RhoB and RhoC in lysates from human monocytes by Western blot analysis. RhoC was detected in a separate blot. Note that the membrane shown on the left was cut (indicated by dashed line) before applying anti-RhoA and anti-RhoB primary antibodies. (**C**) Signaling diagram indicating that the Rho-ROCK (Rho kinase)-nonmuscle myosin II signaling pathway can be blocked at each level by specific inhibitors.

**Figure 2:**
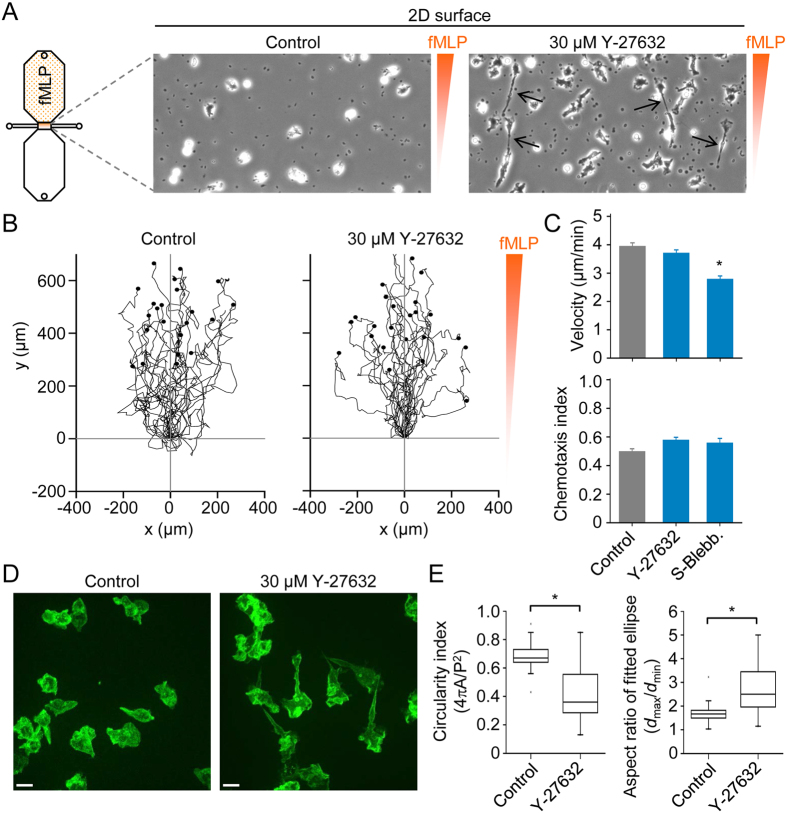
2D chemotaxis assays and effects of ROCK (Rho kinase) and nonmuscle myosin II inhibitors. (**A**) Schematic diagram of a chemotaxis μ-slide, which consists of two 40 μl reservoirs connected by a narrow channel, and phase-contrast snapshots (200 × 400 μm) of cells in the absence (control) or presence of a ROCK inhibitor (Y-27632). The small dark spots are platelets, and the black arrows indicate elongated trailing ends in the presence of Y-27632. (**B**) Migration tracks of monocytes (in a chemotactic fMLP gradient) in the absence of inhibitors (control) or in the presence of Y-27632. Plots were generated by tracking 25 cells (monocytes) and normalizing the start point to x = 0 and y = 0. The y-axis represents the direction of the chemoattractant (fMLP) source. (**C**) Summary data for control, 30 μM Y-27632 and 50 μM S-blebbistatin (S-Blebb.) groups. Data are shown as mean ± s.e.m. for tracked cells (25 per experiment) pooled from three independent experiments for each group. ^*^p < 0.05; Kruskal-Wallis one way analysis of variance on ranks and Dunn’s method for post-hoc comparisons. (**D**) Projected (extended focus) fluorescence images, obtained by spinning disk confocal microscopy, of purified monocytes in the absence (control) or presence of Y-27632. (**E**) Cell shape analysis of control (n = 59) and Y-27632 treated (n = 36) cells. Circularity is a function of the cell perimeter (P) and cell area (A), whereas the aspect ratio is a function of the largest diameter (*d*_max_) and smallest diameter (*d*_min_), after fitting an ellipse to the cell.

Using CD14^+^ cells (monocytes) purified by cell sorting, we could detect mRNA for RhoA, RhoB and RhoC (Fig. 1A). Furthermore, we could detect RhoA, RhoB and RhoC protein using Western blot (Fig. 1B). Thus, unlike mouse macrophages, which only express RhoA and RhoB^14^, human monocytes express all three members of the Rho subfamily. We next set out to explore the effects of pharmacological inhibition of Rho, ROCK and nonmuscle myosin II (NMMII) on monocyte motility and chemotaxis, as indicated in Fig. 1C. Initially, we performed 2D chemotaxis experiments (Fig. 2A), as previously described for mouse macrophages^15^,^16^. However, human monocytes migrated much more rapidly than macrophages (~4 μm/min versus ~1 μm/min), and, thus, we analyzed a shorter time window, 4 h rather than 6 h. Under control conditions, monocytes migrated robustly towards fMLP and mean cell velocity was unaffected in the presence of a ROCK inhibitor (Y-27632) (Fig. 2B,C), but modestly reduced by the nonmuscle myosin II inhibitor S-blebbistatin. Moreover, Y-27632 and S-blebbistatin had no significant effect on chemotaxis efficiency (Fig. 2C). As indicated in Fig. 2A, Y-27632 induced elongated trailing ends in monocytes migrating in a chemotactic gradient. To quantify the effect of Y-27632 on cell morphology, we measured the circularity index and aspect ratio, after fitting an ellipse, of spontaneously migrating cells, imaged by spinning disk confocal microscopy (Fig. 2D,E). Consistent with a more elongated morphology, Y-27632 treatment induced a significantly decreased circularity index, and significantly increased aspect ratio, measured using two-dimensional projections.

### 3D morphology of monocytes migrating on a 2D surface

**Figure 3:**
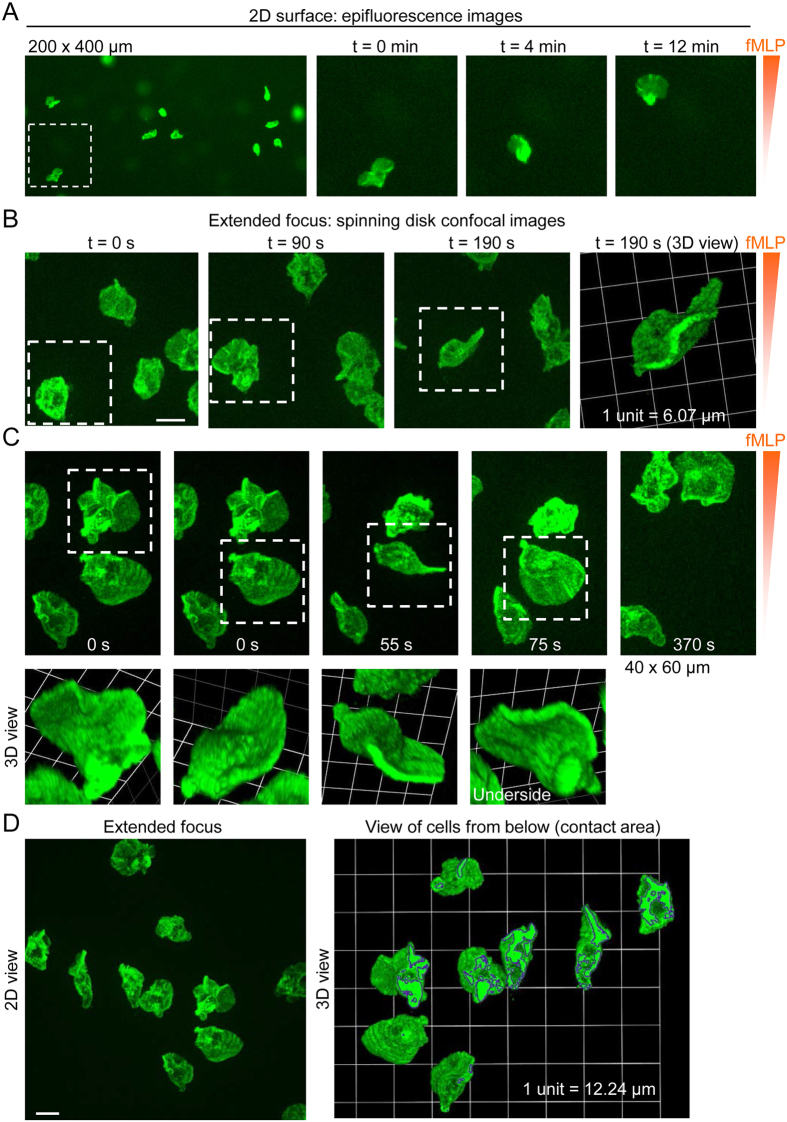
3D morphology of human monocytes migrating on a 2D substrate. (**A**) Snapshot (200 × 400 μm) and enlarged (cropped) time-lapse images (obtained by epi-fluorescence imaging) of human monocytes in a chemotactic fMLP gradient. (**B**) Projected (extended focus) fluorescence images, obtained by time-lapse spinning disk confocal microscopy, of monocytes migrating in an fMLP gradient (scale bar, 10 μm). The white squares track the same cell at different time points, and a 3D view corresponding to t = 190 s is shown on the right. (**C**) Projected (extended focus) fluorescence images (40 × 60 μm; upper panel), obtained by time-lapse spinning disk confocal microscopy, and corresponding reconstruction of 3D morphology (lower panel). (**D**) View of human monocytes from above (2D extended focus view; scale bar, 10 μm) and from below (after rotating the image 180° on the x-axis), showing the cell-substrate contact areas, highlighted by dark boundaries.

To establish a cell-type specific chemotaxis assay which also allows fluorescence imaging, we labeled human monocytes with Alexa Fluor 488-conjugated anti-CD14 antibodies (Fig. 3A). Time-lapse epi-fluorescence imaging provided better visualization of monocytes than phase-contrast imaging, and fluorescent cells appeared to move towards fMLP like fluttering leaves (see supplementary video 1), rather than like crawling macrophages. In sequential snapshots, individual cells appeared to extend one or more broad lamellipodia (generally towards the source of chemoattractant) in one moment and in the next moment exhibited either a compact or comma-shaped morphology (Fig. 3A,B). We used time-lapse spinning disk confocal microscopy to allow rapid confocal sectioning and 3D reconstruction of migrating monocytes (Fig. 3B–D). 3D reconstructed images revealed that, instead of lamellipodia (sheet-like protrusions on a 2D substrate), monocytes generate curved (scalloped) planar protrusions in varying orientations in space (Fig. 3B–D). These 3D membrane dynamics can best be appreciated in videos rather than still images. For example, supplementary video 2 (3D time-lapse image sequence) shows the cells shown in Fig. 3B viewed from above. Supplementary video 3 serves as a (time-lapse 2D imaging) reference sequence for the cells shown in Fig. 3C, whereas supplementary video 4 shows a time-lapse 3D reconstruction of the cells. Classical fibroblast-like lamellipodial protrusions could be detected (not shown), but curved planar protrusions (and membrane undulations) in various orientations predominated. Lack of classical lamellipodia could be appreciated by viewing cells from below (Fig. 3D), which revealed that, typically, only the cell body and the lower edge of planar sheets made contact with the 2D substrate. Supplementary video 5 shows 3D views the same cells (in Fig. 3D) rotated 360° on the x-axis. The low contact area, combined with the reorientations of curved planar protrusions and membrane undulations, may explain the fluttering movement observed in epi-fluorescence time-lapse recordings.

### Phototoxicity and photodegradation of blebbistatin

**Figure 4:**
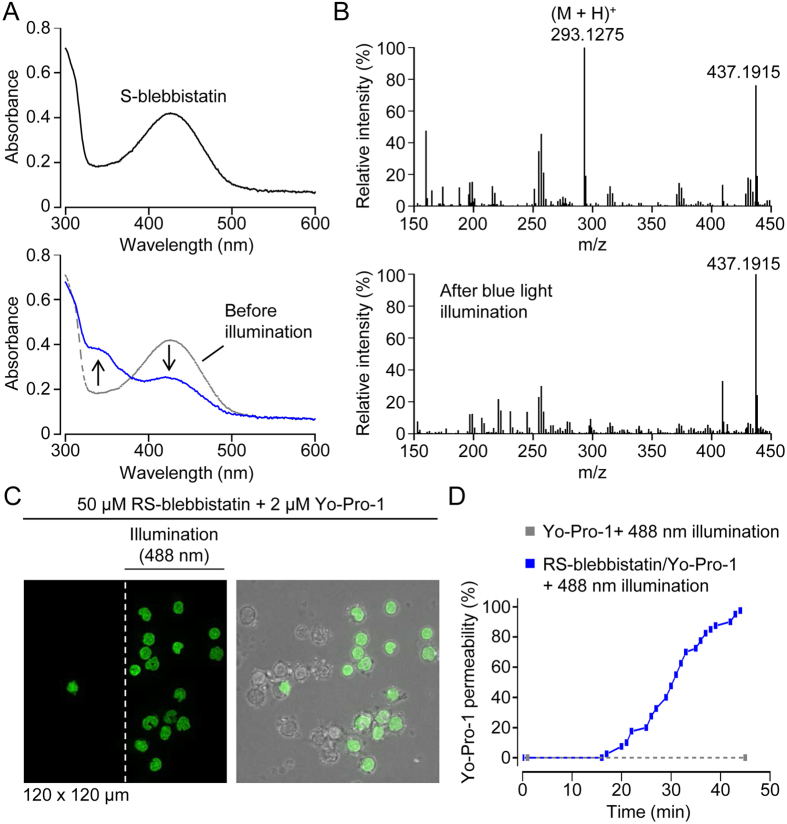
Photodegradation and phototoxicity of S-blebbistatin. (**A**) Absorption scans of S-blebbistatin (50 μM in a 1:1 mixture of DMSO and PBS) before and after 2 h illumination with blue light (450–490 nm). In the lower plot, the absorption scans before (gray; dashed line) and after (blue line) blue light illumination of S-blebbistatin are superimposed. Accordingly, the arrows indicate the change in absorption induced by blue light illumination. (**B**) Mass spectra before and after blue light illumination. The calculated mass of the parent molecule S-blebbistatin ionized by protonation (designated (M + H)^+^) is 293.1275. The m/z ratio of 437.1915 probably corresponds to 2(M - phenyl group) with loss of a methyl group and ionization by Na^+^. (**C**) Fluorescence image (on the left) showing Yo-Pro-1 staining of nuclei, an indicator of loss of plasma membrane integrity, after 45 min intermittent illumination with blue (488 nm) light. The peripheral human mononuclear cells, enriched for monocytes by excessive washing (to remove nonadherent cells), were scanned by serial z-stacks (1 stack = 20 optical slices) obtained at a rate of 4 stacks/min. On the right is an overlay of fluorescence and brightfield images. Note that, after 45 min illumination, the microscope stage was moved to the right to reveal cells (left of the vertical dashed white line) which had not been illuminated. (**D**) Cummulative plot of Yo-Pro-1 permeability in individual cells. In the presence of RS-blebbistatin (solid blue line and dots), the first cell became Yo-Pro-1 positive after 17 min illumination. In the absence of blebbistatin (dashed grey line and dots), blue (488 nm) light illumination did not induce Yo-Pro-1 permeability.

One of the major end targets of Rho-ROCK signaling is nonmuscle myosin II (Fig. 1C), which can be inhibited by blebbistatin^17^, a racemic mixture of R-(+)- and S-(−)-blebbistatin (RS-blebbistatin). In initial chemotaxis assays, we found that illumination of monocytes (labeled with Alexa Fluor 488-conjugated anti-CD14 antibodies) with blue light (excitation: 450–490 nm) in the presence of RS-blebbistatin induced cell death, consistent with earlier reports that blue light photoinactivates blebbistatin and generates cytotoxic intermediates^18^,^19^. Consistent with the data of Kolega^18^, we found that blue light (450–490 nm) illumination induced changes in the absorption spectrum of S-blebbistatin (Fig. 4A), solubilized in a 1:1 mixture (by volume) of DMSO and PBS (phosphate-buffered saline). We extended this work, and found that ionized S-blebbistatin, the active enantiomer, could not be detected by mass spectrometry after blue light illumination, indicating that blebbistatin phototoxicity is associated with photodegradation of the molecule (Fig. 4B).

More recently, the combination of blue light and blebbistatin has been reported to be toxic to human cancer cell lines, and the authors suggested that blebbistatin may be a potential anti-cancer agent^20^. To assess the phototoxicity of blebbistatin in primary human monocytes, we used the cell-impermeant nucleic acid stain Yo-Pro-1 as a probe to detect loss of cell membrane integrity during live-cell recordings (Fig. 4C,D). Using spinning disk confocal microscopy, cells were excessively illuminated with blue (488 nm) light by taking serial z-stacks (20 optical sections of 0.5 μm thickness) at a rate of 4 stacks/min. In the presence of 50 μM RS-blebbistatin, 488 nm illumination induced Yo-Pro-1 staining in more than 95% of cells during the 45 min recording period (Fig. 4C,D). Note that the images shown in Fig. 4C were obtained after moving the microscope stage partially to the right in order to reveal cells (seen on the left half of the field) which were not scanned with 488 nm light; these non-illuminated cells were not used in the data analysis. In the absence of RS-blebbistatin, 488 nm illumination alone was not sufficient to induce Yo-Pro-1 permeability during the 45 min recording period (Fig. 4D).

### Monocyte motility and navigation in loose collagen type I matrices

**Figure 5:**
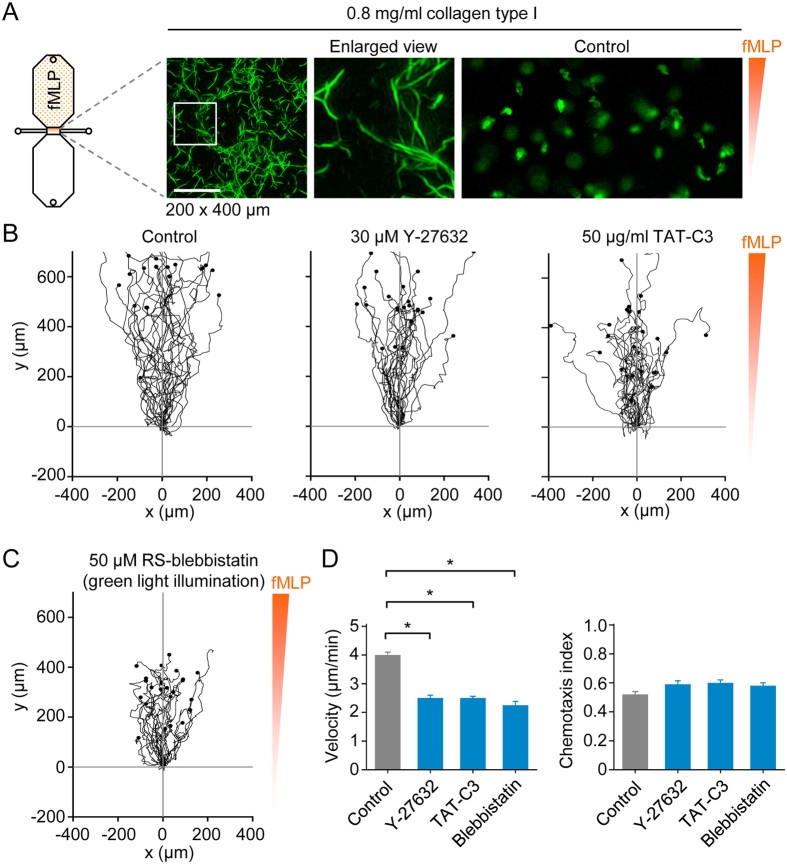
3D chemotaxis assays of monocytes in a loose collagen type I matrix. (**A**) Maximum intensity projection of collagen type I fibers in a loose matrix (0.8 mg/ml collagen), obtained by picrosirius red staining and superresolution structured illumination microscopy, and snapshot (200 × 400 μm) of fluorescently labeled monocytes, in a loose 3D collagen matrix, migrating in a chemotactic fMLP gradient. Scale bar: 10 μm. (**B**) Migration tracks of monocytes (in a loose collagen type I matrix and chemotactic fMLP gradient) in the absence of inhibitors (control) or in the presence of a ROCK inhibitor (Y-27632) or Rho inhibitor (TAT-C3). (**C**) Migration tracks of monocytes (in a loose collagen type I matrix and chemotactic fMLP gradient) in the presence of a nonmuscle myosin II inhibitor (blebbistatin). To circumvent phototoxicity, cells were labeled with a red fluorescent dye and illuminated with green light. (**D**) Summary data. Data are shown as mean ± s.e.m. for tracked cells (25 per experiment) pooled from two to four independent experiments for each group. ^*^p < 0.05; Kruskal-Wallis one way analysis of variance on ranks and Dunn’s method for post-hoc comparisons.

Following the canonical leukocyte adhesion cascade, which involves selectin-mediated tethering and rolling, as well as the activation of integrins, monocytes migrate across the endothelium into the interstitium, a highly heterogeneous 3D environment^21^. Fibrillar collagen, especially type I, is the most abundant extracellular matrix component, and a wide range of lattice densities, from loose to dense, are found in the same tissue^21^, whereas collagen matrices reconstituted *in vitro* exhibit a narrow range. In this study, we tested two collagen type I concentrations, which were intended to represent loose (see Fig. 5A) and dense fibrillar collagen lattices. Monocytes were prestained with fluorescent (Alexa Fluor 488-conjugated) anti-CD14 antibodies (or Alexa Fluor 594-conjugated antibodies, in the case of experiments using blebbistatin) and imaged by time-lapse epi-fluorescence microscopy (Fig. 5A). In a loose collagen matrix (0.8 mg/ml collagen type I), control cells moved robustly towards fMLP (Fig. 5B; supplementary video 6). Monocytes fortuitously in contact with the bottom of the chemotaxis slide were used to set the level of focus, and, thus, most of the time cells moving through the loose matrix appeared as fuzzy (out of focus) objects (supplementary video 6). In the presence of inhibitors of Rho (TAT-C3), ROCKs (Y-27632) or nonmuscle myosin II (blebbistatin), monocyte motility was modestly decreased, but there was no significant impairment in chemotactic navigation (Fig. 5B–D). Interestingly, in parallel experiments, using spinning disk confocal microscopy, we observed that monocytes treated with TAT-C3 developed exceedingly elongated trailing ends when crawling along a 2D surface, but not within a loose 3D matrix (supplementary video 7).

### 3D morphology of monocytes migrating in a loose collagen type I matrix

**Figure 6:**
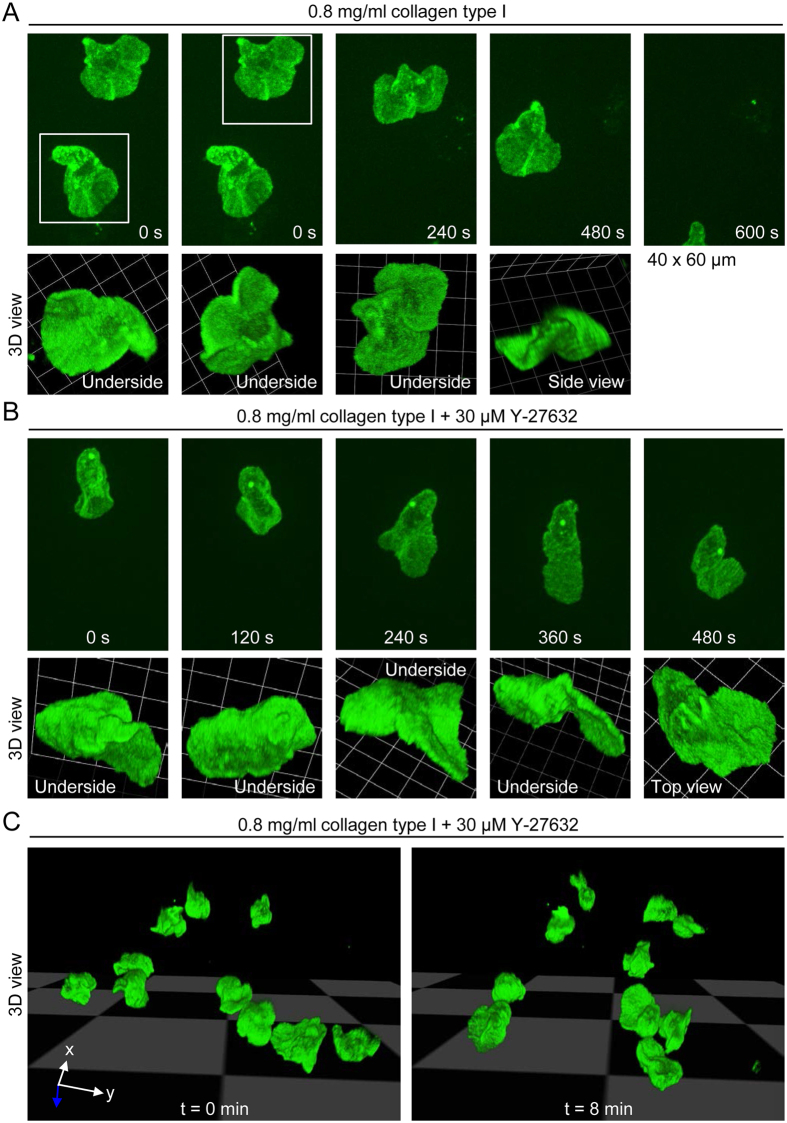
3D morphology of human monocytes migrating in a loose collagen type I matrix. (**A**) Projected (extended focus) fluorescence images (40 × 60 μm; upper panel) of control monocytes migrating in a loose collagen type I matrix (0.8 mg/ml collagen), obtained by time-lapse spinning disk confocal microscopy, and corresponding reconstruction of 3D morphology (lower panel). (**B**) Projected (extended focus) fluorescence images (40 × 60 μm; upper panel) of a monocyte migrating in the presence of the ROCK inhibitor Y-27632, and corresponding reconstruction of 3D morphology (lower panel). (**C**) 3D morphologies of monocytes in the presence of Y-27632 at two time points.

As in the case of 2D migration, we performed spinning confocal microscopy to visualize the mode of migration. Monocytes moved through a loose collagen matrix using curved, planar protrusions in varying orientations (Fig. 6A), as observed for cells moving on a 2D surface. The 3D morphology of the cells shown in Fig. 6A, at t = 0 s, can be nicely seen in supplementary video 8, which shows the cells in different spatial orientations. The mode of migration was not altered in the face of ROCK inhibition (Fig. 6B,C). A time-lapse sequence corresponding to the cell shown in Fig. 6B can be seen in supplementary video 9, and supplementary videos 10, 11 and 12 provide multiple 3D views of the cell at t = 0 s, t = 240 s and t = 360 s, respectively. The time-lapse sequence (supplementary video 9) also shows examples of compact, comma-like and fan-like cell morphologies, when cells are viewed in two dimensions (see also examples in supplementary video 3 and Fig. 3B,C).

### Monocyte motility and navigation in dense collagen type I matrices

**Figure 7:**
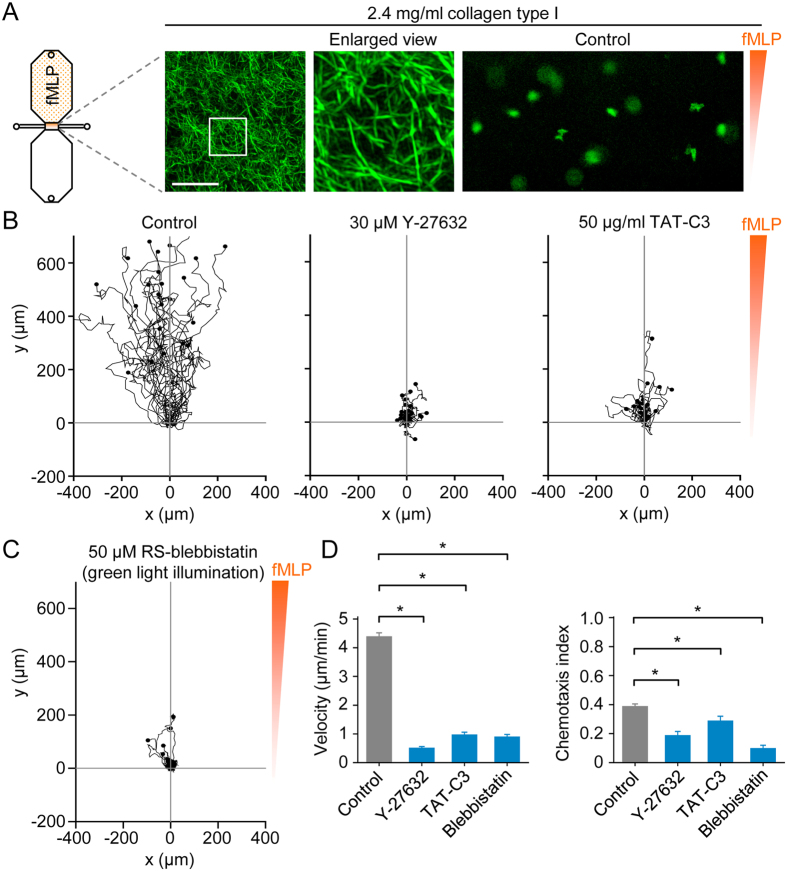
3D chemotaxis assays of monocytes in a dense collagen type I matrix. (**A**) Maximum intensity projection of collagen type I fibers in a dense matrix (2.4 mg/ml collagen), obtained by picrosirius red staining and superresolution structured illumination microscopy, and snapshot (200 × 400 μm) of fluorescently labeled monocytes, in a dense 3D collagen matrix, migrating in a chemotactic fMLP gradient. Scale bar: 10 μm. (**B**) Migration tracks of monocytes (in a dense collagen type I matrix and chemotactic fMLP gradient) in the absence of inhibitors (control) or in the presence of a ROCK inhibitor (Y-27632) or Rho inhibitor (TAT-C3). (**C**) Migration tracks of monocytes (in a dense collagen type I matrix and chemotactic fMLP gradient) in the presence of a nonmuscle myosin II inhibitor (blebbistatin). To circumvent phototoxicity, cells were labeled with a red fluorescent dye and illuminated with green light. (**D**) Summary data. Data are shown as mean ± s.e.m. for tracked cells (25 per experiment) pooled from two to six independent experiments for each group. ^*^p < 0.05; Kruskal-Wallis one way analysis of variance on ranks and Dunn’s method for post-hoc comparisons.

Superresolution structured illumination microscopy showed that polymerized 2.4 mg/ml collagen type I produces a lattice of fibers with small pore sizes (Fig. 7A). In a chemotactic fMLP gradient, fluorescently labeled monocytes robustly migrated through such dense matrices (Fig. 7A,B), whereas motility was profoundly decreased by inhibition of Rho-ROCK signaling (Fig. 7B) or nonmuscle myosin II (Fig. 7C). Surprisingly, the mean velocity of directed monocyte motility in a dense matrix was ~4 μm/min (Fig. 7D), similar to mean values found on a 2D surface (Fig. 2C) or in a loose matrix (Fig. 5D). However, unlike the case for 2D surfaces and loose 3D matrices, inhibition of Rho-ROCK-myosin II signaling impaired chemotactic navigation (Fig. 7D). In relation to this finding, it should be stressed that inhibitors of Rho, ROCK and nonmuscle myosin II frequently caused cells to become trapped in the matrix.

### 3D morphology of monocytes migrating in a dense collagen type I matrix

**Figure 8:**
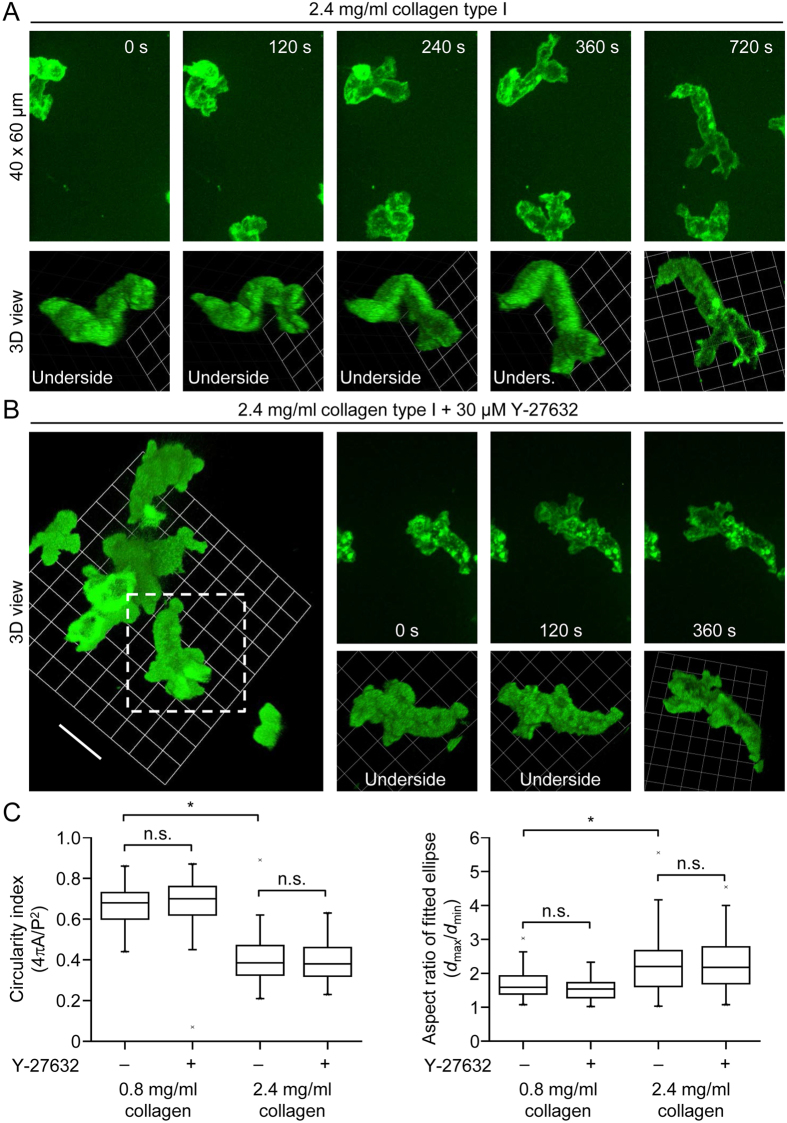
3D morphology of human monocytes migrating in a dense collagen type I matrix. (**A**) Projected (extended focus) fluorescence images (40 × 60 μm; upper panel) of monocytes migrating in a dense collagen type I matrix (2.4 mg/ml collagen), and corresponding reconstruction of 3D morphology (lower panel). (**B**) On the left, 3D views (from above and from the side) of monocytes in a dense matrix (the scale bar to the left of the grid represents 14 μm), and in the presence of the ROCK inhibitor Y-27632. On the right, time-lapse projected (extended focus) fluorescence images (40 × 60 μm; upper panel) of one of the monocytes (indicated on the left image by a white square) migrating in the presence of Y-27632, and corresponding reconstruction of 3D morphology (lower panel). (**C**) Cell shape analysis for the following groups: monocytes without (n = 57) and with 30 μM Y-27632 (n = 67) in a loose matrix (0.8 mg/ml collagen), and monocytes without (n = 68) and with 30 μM Y-27632 (n = 55) in a dense matrix (2.4 mg/ml collagen). Circularity is a function of the cell perimeter (P) and cell area (A), whereas the aspect ratio is a function of the largest diameter (*d*_max_) and smallest diameter (*d*_min_), after fitting an ellipse to the cell. ^*^p < 0.05; Kruskal-Wallis one way analysis of variance on ranks and Dunn’s method for post-hoc comparisons (n.s. = not significant at the 0.05 level of significance).

As in the case for monocyte motility in a loose matrix, we used spinning disk confocal microscopy to determine the morphology of cells moving in a dense matrix (Fig. 8). Under these conditions, monocytes moved in a lobular fashion, such that the front end consisted of blunt protrusions (Fig. 8A), presumably driven by hydrostatic pressure. A time-lapse recording of the cells shown in Fig. 8A can be seen in supplementary video 13, and 3D views of the cells at t = 360 s are provided in supplementary video 14. In the presence of Y-27632, monocytes became stuck in dense matrices, as indicated in Fig. 7B (chemotaxis assays) and Fig. 8B. Three-dimensional views of the cells shown in Fig. 8B, followed by a time-lapse sequence, are shown in supplementary video 15. Thus, Rho-ROCK signaling is critical for motility in a dense matrix, but Rho-ROCK signaling per se does not determine the basic mode of motility, planar protrusions versus lobular protrusions. Indeed, independent of ROCK signaling, the circularity index (measure of roundness) is decreased, and the aspect ratio (measure of elongation) increased, when cells are migrating in a dense matrix, compared to a loose matrix (Fig. 8C).

## Discussion

As dramatically formulated by Couzin-Frankel^22^, “inflammation is a driving force behind chronic diseases that will kill nearly all of us”. Monocytes accumulate at sites of inflammation, as nicely documented by intravital imaging in mouse models^23^,^24^, but in addition to promoting tissue healing and pathogen clearance, these cells may aggravate disease. This “dark side” is particularly apparent in the pathogenesis of atherosclerosis^25^. Here, we established *in vitro* 2D and 3D chemotaxis assays, and used spinning disk confocal microscopy, to investigate the mechanisms of human monocyte motility, and we explored the roles of Rho-ROCK-myosin II signaling on cell shape control, velocity and chemotactic navigation.

Aside from a recent elegant study using a chemoattractant point source (micropipette) and fluorescently labeled chemoattractant proteins^26^, data obtained from real-time monocyte chemotaxis assays are very scarce, as far as we are aware. We used Ibidi 2D chemotaxis μ-slides for both 2D assays, as previously used for mouse macrophages^14^,^16^, and 3D assays, which necessitated addition of the chemoattractant before introduction of the cells. In contrast to resident mouse peritoneal macrophages, freshly isolated human monocytes did not readily flatten onto the ibiTreat (tissue culture treated) surface of μ-slides, and moved like fluttering leaves, with a mean velocity of ~4 μm/min, towards chemoattractant. Under these low adhesion 2D migration conditions, inhibitors of ROCK and nonmuscle myosin II decreased monocyte velocity by ~40%, whereas chemotactic efficiency was unaffected. In macrophages completely lacking the Rho subfamily of GTPases, chemotactic navigation is similarly unaffected, whereas cell velocity is increased by ~50%^14^. Monocytes occasionally developed elongated trailing ends, but much less dramatically than pan-Rho knockout mouse macrophages^14^ or macrophages treated with ROCK inhibitors^15^, presumably reflecting weak (monocytes) versus strong (macrophages) cell-substrate adhesions. Notably, in the presence of inhibitors of Rho-ROCK-myosin II, elongated trailing ends were not observed in cells migrating in loose or dense collagen type I matrices, except when cells transiently came in contact with the floor of the chemotaxis slide. This reinforces the notion that strong integrin-mediated adhesions are required for Rho-ROCK inhibitors to induce the development of elongated trailing ends in migrating cells.

In single frames of time-lapse epi-fluorescence recordings, human monocytes typically exhibited one of three basic morphologies (compact, fan-like or comma-like), and in time-lapse videos the cells appeared to flutter like leaves towards chemoattractant. Indeed, 3D reconstructions of z-stacks obtained by sequential spinning disk confocal microscopy indicated that the cells generated leaf blade-like protrusions tilted away from the x-y plane (corresponding to fan-like morphologies in x-y projection views) or rotated towards an orthogonal plane (corresponding to comma-shaped cells in x-y projection views). In other words, on uncoated 2D plastic surfaces, monocytes move using highly dynamic, three-dimensional protrusive structures, rather than crawling along the 2D substrate using lamellipodia. This raises the question whether monocytes can swim, as demonstrated for *Dictyostelium* amoebae and neutrophils in experiments using a point source of chemoattractant and isodense media^27^. In favor of this possibility, monocytes undergo rapid cell shape changes, and the pushing of curved planar protrusions against fluid, as well as membrane undulations, may support swimming.

We used superresolution structured illumination microscopy, as well as confocal reflection microscopy (not shown), to confirm that the collagen type I used in our studies formed fibrillar lattices. The loose collagen matrix exhibited more heterogeneous pore sizes, such that cells would be expected to encounter both low- to high-density fibrillar regions, as found *in vivo*^21^,^28^. In both loose and dense fibrillar collagen matrices, monocytes moved rapidly along fMLP gradients, as in the case of 2D assays, whereas inhibition of Rho-ROCK-myosin II signaling modestly decreased velocity in a loose matrix, but severely impaired motility in a dense matrix. As in the case of 2D motility, spinning disk confocal imaging revealed that monocytes moved in a loose matrix using curved planar protrusions. In contrast, in a dense matrix, virtually all cells moved in a sausage-like (or worm-like) fashion, involving blunt lobular protrusions. The high sensitivity to inhibition of Rho-ROCK-myosin II signaling suggests that monocytes switch from planar protrusion (pseudopodia) mediated motility to pressure (hydraulic) driven motility when the matrix pores becomes tight. Thus, human monocytes, like mouse dendritic cells^6^, use Rho-ROCK-myosin II signaling to “squeeze” through tight spaces. How a monocyte senses that it is in a tight matrix is unclear. One possibility is that membrane tension, the “sensor” for being in a tight matrix, is generated when the cell body becomes stuck. Such a sensor has recently been suggested to suppress lamellipodial membrane extensions on the trailing edge of migrating cells^29^. G_12_/G_13_-coupled receptors provide a potential link between membrane tension and Rho activity, although, at least on 2D surfaces, G_12_/G_13_ double knockout macrophages do not develop elongated trailing ends (unpublished data) in contrast to pan-Rho-deficient cells^15^.

In conclusion, monocytes have been neglected in real-time imaging studies of motility and chemotaxis, although these cells play a pivotal role in human disease. We used 2D and 3D chemotaxis assays, as well sequential high-resolution 3D imaging, to better understand how human monocytes move and navigate, and to elucidate the roles of Rho-ROCK-myosin II signaling. In all conditions tested (2D substrate, 3D loose matrix and 3D dense matrix), monocytes moved with modestly high velocity and efficient chemotactic navigation, although the transition from loose to dense matrix caused the cells to switch from a fluttering leaf-like mode to a highly Rho-ROCK-myosin II-dependent lobular mode. Notably, the 3D membrane dynamics underlying the so-called fluttering leaf-like mode of motility could not be predicted from x-y projection views, which stresses the importance of sequential 3D (4D) imaging for understanding how cells move. It would be conceptually useful to investigate whether monocytes can swim in an isodense medium.

## Materials and Methods

### Materials

Recombinant TAT-C3 was prepared as previously described^30^, and used at a concentration of 50 μg/ml. TAT-C3 is a cell permeable form of exoenzyme C3 transferase (from *Clostridium botulinum*), a Rho-specific inhibitor. It consists of C3 transferase fused to the protein transduction domain TAT (trans-activator of transcription), encoded by the human immunodeficiency virus gene *Tat*^31^. Y-27632 (Sigma-Aldrich, Germany), dissolved in DMSO to give a 30 mM stock solution, was used at 30 μM. Racemic RS-blebbistatin, R-(+)-blebbistatin and S-(−)-blebbistatin were obtained from Tocris Bioscience (Bristol, United Kingdom) and dissolved in anhydrous dimethyl sulfoxide (DMSO) to yield 50 mM stock solutions.

### Isolation of human peripheral blood mononuclear cells

Peripheral blood (~15 ml) was collected into 2 × 7.5 ml S-Monovette tubes (Sarstedt, Nümbrecht, Germany) via a 21G Venoflex venipuncture kit (B. Braun Melsungen, Melsungen, Germany), and mixed 1:1 with Dulbecco’s phosphate buffer saline (PBS; Sigma-Aldrich, Germany). The diluted blood was gently layered on top of 15 ml Histopaque-1077 (1.077 g/ml; 10771, Sigma-Aldrich, Germany) in a 50 ml plastic tube (Sarstedt, Nümbrecht, Germany) and subsequently centrifuged using the following settings of an Eppendorf centrifuge 5804 R (Hamburg, Germany): rotational speed, 1800 rpm (revolutions per minute); time, 20 min; acceleration ramp, 4 (range, 0–9) and deceleration ramp, 0 (range, 0–9). Peripheral blood mononuclear cells, the ring of cells immediately above the density gradient medium, were isolated using a pipette and washed. The cells were, when required, incubated with Alexa Fluor 488-conjugated anti-human CD14 antibodies (dilution, 1:40; clone M5E2, catalog number 301811, BioLegend (Fell, Germany) for 15 min. After washing and centrifugation (300 × g for 7.5 min), the pellet was resuspended in RPMI 1640 medium containing 20 mM Hepes (Biochrom, Berlin, Germany). Alternatively, monocytes were labeled with phycoerythrin-conjugated anti-human CD14 antibodies (dilution 1:40; clone M5E2, catalog number 555398; Becton Dickinson, Heidelberg, Germany) or Alexa Fluor 594-conjugated anti-human CD14 antibodies (dilution, 1:40; clone HCD14, catalog number 325630, BioLegend (Fell, Germany). In selected experiments, human monocytes were purified by negative selection using the Monocyte Isolation Kit II from Miltenyi Biotec (Bergisch Gladbach, Germany).

### Ethics statement

Methods were carried out in accordance with the approved guidelines of the University of Münster. All experimental protocols were approved by the local ethics committee of the University of Münster. Informed consent was obtained for the isolation of peripheral venous blood (15 ml) from donors.

### 2D chemotaxis assays

Chemotaxis assays were performed as previously described^14^,^32^, except that cells were allowed 2 h, rather than 3 h, to adhere to the narrow channel (observation area) connecting the two 40 μl reservoirs of the uncoated (ibiTreat) μ-slide chemotaxis chamber (Ibidi, Martinsried, Germany). Chemotaxis slides were gently filled with medium by rotating the volume adjustment sleeve of Eppendorf Research Plus pipettes (2–20 μl and 10–100 μl). After filling, 15 μl medium containing fMLP (formyl-Met-Leu-Phe) and Patent Blue V was drawn into one of the two 40 μl reservoirs. After diffusion in the reservoir, the final concentration of fMLP was 20 nM. Patent Blue V (final concentration, 0.003%) served as a visual indictor of gradient formation.

The observation area was imaged by phase-contrast and/or fluorescence microscopy (excitation: 450–490 nm; emission: 500–550 nm) via a 10x/0.3 objective lens. In the case of experiments using blebbistatin, blue light illumination (450–490 nm) induced cell death. To avoid blebbistatin-mediated phototoxicity, we switched to Alexa Fluor 594-conjugated anti-CD14 antibody labeling and excited cells with green light (excitation: 532–558 nm; emission: 570–640 nm), which is not absorbed by the drug. Images were captured every 2 min for 12 h, and cell migration tracks between 3 and 7 h were analyzed with ImageJ (National Institutes of Health) using a manual tracking plugin and the chemotaxis and migration tool from Ibidi. Twenty-five randomly selected cells (monocytes) were manually tracked in each chemotaxis experiment. The y-forward migration (chemotaxis) index was used as a measure of chemotactic efficiency. The index is obtained by dividing the net displacement along the y-axis by the total distance (in the x-y plane) migrated by the cell (values range from −1 to +1).

Experiments were performed on the stage of an inverted microscope (Axio Observer, Zeiss) equipped with a temperature-controlled incubator (incubator XL S, Zeiss). The temperature was maintained at 37 °C.

### 3D chemotaxis assays

Rat tail collagen, type I (BD (Becton Dickinson) Biosciences, Heidelberg, Germany), which had not been treated with the protease pepsin, was initially diluted to 4.8 or 1.6 mg/ml using pH neutralizing solution, then further diluted 1:1 (v/v) with monocyte-labeled mononuclear cell suspension to yield collagen concentrations of 2.4 or 0.8 mg/ml, respectively. To prepare 1.6 mg/ml collagen, 100 μl collagen type I (10.21 mg/ml in 0.02 N HCl) was diluted with 538.1 μl medium, consisting of 100 μl RPMI 1640 Hepes medium (2x concentrated), 436.1 μl RPMI 1640 Hepes medium (1x) and 2 μl 1 N NaOH. Alternatively, to prepare 4.8 mg/ml collagen, 100 μl collagen type I (10.21 mg/ml in 0.02 N HCl) was diluted with 112.7 μl medium, prepared by combining 100 μl RPMI 1640 Hepes medium (2× concentrated), 10.7 μl RPMI 1640 Hepes medium (1×) and 2 μl 1 N NaOH. The pH of the 2× concentrated collagen solutions was 7.2–7.4, assessed using pH-indicator strips (pH 6.5–10.0; MColorpHast, Merck KGaA, Darmstadt, Germany), which we designated as (physiologically) pH neutralized.

Typically, 100 μl cell suspension was mixed with 100 μl of the 2x concentrated (and pH neutralized) collagen solution. Next, 8 μl of the cell suspension was drawn into the narrow (1000 μm × 2000 μm × 70 μm) channel of a prefilled and uncoated (ibiTreat) μ-slide chemotaxis chamber (Ibidi) using an Eppendorf Research Plus (2–20 μl) pipette. Immediately before adding the cell suspension, chemoattractant (fMLP) was drawn into one of the two reservoirs.

### Cell sorting and RT-PCR analyses

Peripheral blood mononuclear cells were incubated with Alexa Fluor 488-conjugated anti-human CD14 antibodies, washed and resuspended in AutoMACS running buffer (Miltenyi Biotec), which contained 2 mM EDTA and 0.5% bovine serum albumin in PBS (pH 7.2). CD14^+^ cells were isolated using a BD FACSAria II flow cytometer (BD Biosciences, San Jose, CA), and diluted in RPMI 1640 medium containing 20 mM Hepes, 10% heat-inactivated FCS, 100 U/ml penicillin and 100 μg/ml streptomycin. Total RNA was isolated using a RNeasy Mini Kit from Qiagen (Hilden, Germany). cDNA was synthesized using SuperScript III reverse transcriptase (Invitrogen, Germany). The thermocycling protocol for the PCR was as follows: 94 °C for 4 min, then 29 cycles of 94 °C for 30 s, 60 °C for 30 s, and 72 °C for 45 s. The following primers, as described by Fritz *et al*.^33^ (except for changes indicated by underline), were used (product sizes shown in parentheses): RhoA (582 bp): forward, ATGGCTGCCATCCGGAAGAAA; reverse: TCACAAGACAAGGCACCCAGA; RhoB (548 bp): forward, GCGTGTGGCAAGACGTGCC; reverse: TCATAGCACCTTGCAGCAGTT; RhoC (582 bp): forward, ATGGCTGCAATCCGAAAGAAG; reverse, TCAGAGAATGGGACAGCCCCT.

### Western blot analyses

Monocytes were lysed in buffer containing 100 mM NaCl, 2 mM MgCl_2_, 1 mM dithiothreitol, 1% Nonidet P-40, 10% glycerol, 5 mM NaF, 1 mM Na_3_VO_4_ (sodium orthovanadate), the protease inhibitors leupeptin, aprotinin and pefabloc (each at 10 μg/ml), and 50 mM Tris-HCl (pH 7.4). Proteins were separated by 15% SDS-PAGE (sodium dodecyl sulfate polyacrylamide gel electrophoresis) and transferred onto polyvinylidene difluoride membranes (Roche, Mannheim, Germany). Membranes were blocked for 1 h at room temperature in TBS containing 5% bovine serum albumin and 0.1% Tween 20, followed by overnight incubation (4 °C) with the following primary antibodies: mouse monoclonal anti-RhoA (26C4 (sc-418), Santa Cruz Biotechnology, Heidelberg, Gerrmany), rabbit polyclonal anti-RhoB (119 (sc-180), Santa Cruz Biotechnology) and rabbit monoclonal anti-RhoC (D40E4) (#3430, New England Biolabs (Cell Signaling Technology), Frankfurt am Main, Germany). For detection, horseradish peroxidase-conjugated secondary antibodies (Dianova, Hamburg, Germany) were used in combination with SuperSignal West Pico chemiluminescence substrate (Perbio, Bonn, Germany).

### Spinning disk confocal microscopy

3D images of living monocytes migrating on a 2D surface or in a collagen matrix were obtained using an UltraVIEW Vox 3D live cell imaging system (Perkin Elmer, Rodgau, Germany) coupled to a Nikon Eclipse Ti inverse microscope. The system incorporated a Yokogawa (Japan) CSU-X1 spinning disk scanner, a Hamamatsu (Japan) C9100–50 EM-CCD camera (1000 × 1000 pixels) and Volocity software. Cells were imaged via a Nikon CFI (chromatic aberration-free infinity) Apo TIRF 60x (NA 1.49) oil immersion objective lens, which has a (coverslip corrected) working distance of 0.12 mm. The temperature was maintained at 37 °C using an Okolab all-in-one stage incubator (Okolab, Ottaviano, Italy). Z-stacks of monocytes labeled with Alexa Fluor 488-conjugated anti-human CD14 antibodies were taken every 5 s or 15 s. To reduce phototoxicity during time-lapse spinning disk confocal microscopy experiments, the medium was supplemented with the reactive oxygen species scavenger N-(2-mercaptoproprionyl)-glycine (1 mM).

### Superresolution structured illumination microscopy of 3D collagen matrices

Collagen was stained with picrosirius red using a kit from Polysciences Europe (Eppelheim, Germany). Picrosirius red stains type I and Type III collagen, and stained collagen can be imaged by polarization^34^ or fluorescence microscopy^35^. 3D images were obtained using an ELYRA S.1 superresolution structured illumination microscopy (SR-SIM) system (Zeiss, Germany), which provides resolution beyond the diffraction limit^36^.

### Absorption and fluorescence spectroscopy

Absorption and fluorescence spectra of 50 μM or 500 μM S-blebbistatin dissolved in DMSO were obtained using a multimode microplate reader (Infinite 200 PRO, Tecan, Salzburg, Austria). In addition, 100 μl of 50 μM S-blebbistatin, dissolved in a 1:1 mixture of DMSO and PBS, was pipetted into a UVette (Eppendorf disposable cuvette (50–2000 μl); Eppendorf, Hamburg, Germany) and illuminated on the stage of an inverted microscope with blue light (450–490 nm) via a 10x/0.3 objective lens. Absorption spectra before and after blue light illumination were recorded using a UviLine 9400 spectrometer (Schott Instruments, Germany).

### Mass spectrometry

Fourier transform mass spectrometry (also known as Fourier transform ion cyclotron mass spectrometry) was performed using an LTQ Orbitrap XL mass spectrometer (Thermo Scientific, Dreieich, Germany) and heated electrospray ionization in positive ion mode. Before analysis, samples (50 μM S-blebbistatin before and after blue light illumination) were diluted to a final concentration of 5 μM using methanol. Data were analyzed using Xcalibur 2.07 software (Thermo Scientific).

### Statistical analysis

Two or more independent experiments were performed for each group in a data set. Normality and homoscedasticity were tested using the Shapiro-Wilk and Levene tests, respectively. A one-way ANOVA (analysis of variance) was used to test for statistical differences at the 0.05 level of significance. When the assumed conditions of normality and homogeneity of variance were not fulfilled, as in most cases, we used the non-parametric Kruskal-Wallis one way analysis of variance on ranks (at the 0.05 level of significance). Post-hoc multiple comparisons were made using Dunn’s method. Statistical analyses were performed using Origin 2015 SR2 or SigmaPlot (version 12) software (Systat Software, Erkrath, Germany), and data are presented as mean ± standard error (s.e.m.) or blox plots.

## Additional Information

**How to cite this article**: Bzymek, R. *et al*. Real-time two- and three-dimensional imaging of monocyte motility and navigation on planar surfaces and in collagen matrices: roles of Rho. *Sci. Rep*. **6**, 25016; doi: 10.1038/srep25016 (2016).

## Supplementary Material

Supplementary Information

Supplementary Video S1

Supplementary Video S2

Supplementary Video S3

Supplementary Video S4

Supplementary Video S5

Supplementary Video S6

Supplementary Video S7

Supplementary Video S8

Supplementary Video S9

Supplementary Video S10

Supplementary Video S11

Supplementary Video S12

Supplementary Video S13

Supplementary Video S14

Supplementary Video S15
