# Supplementary material for: Real-time two- and three-dimensional imaging of monocyte motility and navigation on planar surfaces and in collagen matrices: roles of Rho
Source: Sci Rep. 2016 Apr 28;6:25016. doi: 10.1038/srep25016 (PMC4848558; doi:10.1038/srep25016)
Supplement: Supplementary Information [file srep25016-s1.pdf]

Real-time two- and three-dimensional imaging of monocyte motility and navigation on planar surfaces and in collagen matrices: roles of Rho

Robert Bzymek[1], Markus Horsthemke[2], Katrin Isfort[1], Simon Mohr[1], Kerstin Tjaden[3], Carsten Müller-Tidow[4], Marlies Unterberg[5], Tanja Schwerdtle[6], Martin Bähler[2], Albrecht Schwab[1] & Peter J. Hanley[2]

Legends for supplementary videos

Supplementary video 1. Human monocyte-specific real-time 2D chemotaxis assay. The monocytes seen migrating in a chemotactic fMLP gradient were pre-labeled with fluorescent (Alexa Fluor 488-conjugated) anti-CD14 antibodies. The time-lapse images are 400 x 600 µm and cover a time span of 4 h (60 frames are shown at a rate of 10 frames per second).

Supplementary video 2. Time-lapse 3D reconstructed images, acquired by spinning disk confocal microscopy, of human monocytes migrating on a 2D surface. Image stacks (14 z-slices with 1.12 µm steps) were captured at a rate of 12 timepoints/min via a Nikon 60x (numerical aperture, 1.49) oil-immersion objective lens. The recording represents 20 min. The orientation arrows show x, -y and -z.

Supplementary video 3. Time-lapse (extended focus) images of human monocytes migrating on a 2D surface. Image stacks (14 z-slices with 1.45 µm steps) were captured at a rate of 12 timepoints/min, and the recording represents 20 min.

Supplementary video 4. Time-lapse 3D reconstructed images, acquired by spinning disk confocal microscopy, of human monocytes migrating on a 2D surface. Image stacks (14 z-slices with 1.45 µm steps) were captured at a rate of 12 timepoints/min, and the recording represents 20 min. The orientation arrows show x, -y and -z.

Supplementary video 5. 3D reconstructed images, rotated 360° on the x-axis, of human monocytes migrating on a 2D surface. The orientation arrows show x, -y and -z.

Supplementary video 6. Human monocyte-specific real-time 3D chemotaxis assay using a low density collagen (0.8 mg/ml) matrix. The monocytes seen migrating in a chemotactic fMLP gradient were pre-labeled with fluorescent (Alexa Fluor 488-conjugated) anti-CD14 antibodies. The time-lapse images are 400 x 600 µm and cover a time span of 4 h (60 frames are shown at a rate of 10 frames per second).

Supplementary video 7. 3D reconstruction of TAT-C3 (50 µg/ml; Rho inhibitor) treated human monocytes (labeled with Alexa Fluor 488-conjugated anti-CD14 antibodies) migrating in a low density collagen (0.8 mg/ml) matrix. The image was acquired by spinning disk confocal microscopy, using a Nikon 60x (numerical aperture, 1.49) oil-immersion objective lens and 67 z-slices (0.5 µm step size). The orientation arrows show x, -y and -z.

Supplementary video 8. 3D reconstruction of human monocytes (labeled with Alexa Fluor 488-conjugated anti-CD14 antibodies) migrating in a low density collagen (0.8 mg/ml) matrix. The image was acquired by spinning disk confocal microscopy, using a Nikon 60x (numerical aperture, 1.49) oil-immersion objective lens and 23 z-slices (1 µm step size). The orientation arrows show x, -y and -z.

Supplementary video 9. Time-lapse (extended focus) images of a Y-27632 (30 µM; Rho kinase inhibitor) treated human monocyte (labeled with Alexa Fluor 488-conjugated anti-CD14 antibodies) migrating in a low density collagen (0.8 mg/ml) matrix. Images (17 z-slices at 1 µm step size) were acquired at a rate of 0.5 timepoints/min and the movie represents a recording time of 26 min.

Supplementary video 10. 3D reconstruction of a Y-27632 (30 µM) treated human monocyte (labeled with Alexa Fluor 488-conjugated anti-CD14 antibodies) migrating in a low density collagen (0.8 mg/ml) matrix. The image was acquired by spinning disk confocal microscopy, using a Nikon 60x (numerical aperture, 1.49) oil-immersion objective lens and 17 z-slices (1 µm step size). The orientation arrows show x, -y and -z.

Supplementary video 11. 3D reconstruction of a Y-27632 (30 µM) treated human monocyte (labeled with Alexa Fluor 488-conjugated anti-CD14 antibodies) migrating in a low density collagen (0.8 mg/ml) matrix. The image was acquired by spinning disk confocal microscopy, using a Nikon 60x (numerical aperture, 1.49) oil-immersion objective lens and 17 z-slices (1 µm step size). The orientation arrows show x, -y and -z.

Supplementary video 12. 3D reconstruction of a Y-27632 (30 µM) treated human monocyte (labeled with Alexa Fluor 488-conjugated anti-CD14 antibodies) migrating in a low density collagen (0.8 mg/ml) matrix. The image was acquired by spinning disk confocal microscopy, using a Nikon 60x (numerical aperture, 1.49) oil-immersion objective lens and 17 z-slices (1 µm step size). The orientation arrows show x, -y and -z.

Supplementary video 13. Time-lapse (extended focus) images of a human monocyte (labeled with Alexa Fluor 488-conjugated anti-CD14 antibodies) migrating in a high density collagen (2.4 mg/ml) matrix. Image stacks (42 z-slices at 1 µm step size) were acquired by spinning disk confocal microscopy at a rate of 0.5 timepoints/min. The movie represents a recording time of 30 min.

Supplementary video 14. 3D reconstructions of human monocytes (labeled with Alexa Fluor 488-conjugated anti-CD14 antibodies) migrating in a high density collagen (2.4 mg/ml) matrix. The image

was acquired by spinning disk confocal microscopy, using a Nikon 60x (numerical aperture, 1.49) oil-immersion objective lens and 42 z-slices (1 µm step size). The orientation arrows show x, -y and -z.

Supplementary video 15. 3D reconstructions of Y-27632 (30 µM) treated human monocytes (labeled with Alexa Fluor 488-conjugated anti-CD14 antibodies) migrating in a high density collagen (2.4 mg/ml) matrix. The image was acquired by spinning disk confocal microscopy, using a Nikon 60x (numerical aperture, 1.49) oil-immersion objective lens and 44 z-slices (1 µm step size). The orientation arrows show x, -y and -z.
